# Biased Coupling to β-Arrestin of Two Common Variants of the CB_2_ Cannabinoid Receptor

**DOI:** 10.3389/fendo.2021.714561

**Published:** 2021-08-16

**Authors:** Gábor Turu, Eszter Soltész-Katona, András Dávid Tóth, Cintia Juhász, Miklós Cserző, Ádám Misák, András Balla, Marc G. Caron, László Hunyady

**Affiliations:** ^1^Department of Physiology, Faculty of Medicine, Semmelweis University, Budapest, Hungary; ^2^MTA-SE Laboratory of Molecular Physiology, Hungarian Academy of Sciences and Semmelweis University, Budapest, Hungary; ^3^Department of Cell Biology, Duke University Medical Center, Duke University School of Medicine, Durham, NC, United States

**Keywords:** polymorphisms, biased, signaling, β-arrestin2, Q63R, L133I, CB2R, CB_2_ cannabinoid receptor

## Abstract

β-arrestins are partners of the G protein-coupled receptors (GPCRs), regulating their intracellular trafficking and signaling. Development of biased GPCR agonists, selectively targeting either G protein or β-arrestin pathways, are in the focus of interest due to their therapeutic potential in different pathological conditions. The CB_2_ cannabinoid receptor (CB_2_R) is a GPCR involved in various functions in the periphery and the central nervous system. Two common occurring variants of CB_2_R, harboring Q63R or L133I missense mutations, have been implicated in the development of a diverse set of disorders. To evaluate the effect of these mutations, we characterized the binding profile of these mutant CB_2_ receptors to G proteins and β-arrestin2. Although their ability to inhibit cAMP signaling was similar, the Q63R mutant had increased, whereas the L133I mutant receptor had decreased β-arrestin2 binding. In line with these observations, the variants also had altered intracellular trafficking. Our results show that two common variants of the CB_2_ receptor have biased signaling properties, which may contribute to the pathogenesis of the associated disorders and may offer CB_2_R as a target for further development of biased receptor activation strategies.

## Introduction

The two major known receptors for exogenous and endogenous cannabinoids are the CB_1_ and CB_2_ cannabinoid receptors (CB_1_R and CB_2_R), they belong to the G protein-coupled receptor (GPCR) superfamily (1). Both cannabinoid receptors are coupled to G_i/o_ proteins, which inhibit adenylyl cyclase activity, activate voltage-gated calcium channels, initiate mitogen-activated protein kinase (MAPK) and phosphoinositide 3-kinase (PI3K)-Akt pathways (2–4).

CB_2_R is abundantly expressed in peripheral organs with important functions in immune cells (5). Beyond the receptors’ peripheral expression, it may also play an important role in the regulation of the central nervous system, as well. Although CB_2_R is expressed at low levels in the brain under physiological conditions, it is upregulated in various pathological conditions (6) and plays a role in some mental disorders such as schizophrenia (7, 8), depression, or alcoholism (9, 10).

CB_2_R, like the vast majority of GPCRs, binds β-arrestin proteins and internalizes upon stimulation (11–13). The C-terminus of the agonist-bound receptor is phosphorylated by G protein-coupled receptor kinase (GRK) proteins, a process that triggers the recruitment of β-arrestins to the receptor (14). In addition to desensitization and internalization of the receptors, β-arrestin proteins play a role in initiating further signaling pathways in the cell. They act as scaffold proteins that trigger a wide range of signaling events, such as the mitogen-activated protein kinase (MAPK) pathway (15–17). In this way, they regulate the growth of cells, play a role in the regulation of pathways involved in cell survival, growth, apoptosis, and modulation of immune function. Manipulation of their functions may be beneficial in inflammatory diseases, fibrosis, and cancer (18–20). Ligands selectively targeting either β-arrestin or G protein activation, called biased ligands, are being developed and have been shown to be beneficial in various disorders (21–24). In the case of the CB_2_Rs, many agonists are biased in one or the other direction (13, 25–27).

In recent years, the importance of polymorphisms of the human gene of CB_2_R has emerged in several psychiatric disorders. One missense polymorphism is the AA-GG conversion at positions 188-189 of the CB_2_R coding DNA, (rs2501432) which causes a glutamine-arginine amino acid change at position 63 of the protein (CB_2_R-Q63R). This mutation allele frequency seems to be 65% worldwide (28), and has been suggested to affect some conditions like depression, alcoholism (9, 10), schizophrenia (8), autoimmune diseases (29, 30), juvenile idiopathic arthritis (31), immune thrombocytopenic purpura in children (32, 33), and others. In the case of another missense polymorphism (rs41311993), which involves a leucine-isoleucine exchange at position 133 (CB_2_R-L133I), a significantly higher mutant allele frequency was found in bipolar disorder patients in an Italian population sample (34). rs413119933 SNP was detected in Italy at the highest rate with a prevalence of 2% (28).

The exact mechanism, by which these polymorphisms affect the function of the CB_2_R, is still poorly understood. The aim of this study was to investigate the impact of the naturally occurring mutations, CB_2_R-Q63R and CB_2_R-L133I, on the G protein activation, β-arrestin binding, cellular distribution, and internalization of CB_2_R.

## Materials and Methods

### Materials and Plasmid DNA Constructs

Molecular biology reagents and High Capacity NeutrAvidin-Agarose Resin were from Thermo Scientific (Waltham, MA). 2-Arachidonylglycerol (2-AG) and JWH-133 were from Tocris. Cell culture reagents were from Invitrogen and Biosera. Coelenterazine *h* was obtained from Regis Technologies (Morton Grove, IL). Biotin was from SERVA Electrophoresis GmbH (Heidelberg, Germany).

The pRluc8-N1, MP–mVenus, βarr2–Venus, Venus–β1 and γ2 plasmids were described previously (35, 36). pBirA-R118G-N1 vector was created by replacing the Rluc8 sequence in pRluc8-N1 vector with the BirA-R118G sequence with AgeI/NotI restriction enzymes after its PCR amplification from pcDNA3.1-BirA-R118G plasmid [acquired from Addgene (37)]. The plasmid coding human CB_2_R was from cDNA Resource Center (Bloomsberg, PA). We introduced the Q63R and L133I mutations into CB_2_R with precise gene fusion PCR. To generate wild-type or mutant forms of CB_2_R–Rluc8, CB_2_R–YFP, and CB_2_R–BirA-R118G, we amplified the coding sequence of CB_2_R without stop codon and inserted it into pRluc8-N1, pEYFP-N1, or pBirA-R118G-N1 vectors, respectively. βarr2–Rluc8 was created by replacing Venus to Rluc8 in βarr2–Venus between AgeI/NotI restriction sites in Clontech N1 vector. To generate Gα_i1_–Rluc8, we inserted Rluc8 with linkers (SGGGGS) between the 91^st^ and the 92^nd^ residues of Gα_i1_ as in a previous study (38). β2-adaptin–Venus was generated by N-terminally fusing the β1 subunit of adaptor-related protein complex 2 to Venus in pVenus-N1. Venus–Rab4, Venus–Rab5, and Venus–Rab11 were created by replacing EYFP to monomeric Venus in YFP–Rab4, YFP–Rab5, YFP–Rab11 constructs (39).

### Cell Culture and Transfection

HEK 293T cells were purchased from the American Type Culture Collection (ATCC CRL-3216) and were cultured in DMEM medium supplemented with 10% fetal bovine serum (FBS) and 1% penicillin/streptomycin (Invitrogen) in 5% CO_2_ atmosphere at 37°C. For BRET measurements, cells were transfected in suspension using Lipofectamine 2000 (Invitrogen) according to the manufacturer’s protocol and plated on white poly-L-lysine coated 96-well plates. For the other experiments, we used the calcium phosphate precipitation method either with adherent cells or in cell suspension. Briefly, plasmid DNAs were mixed in sterile distilled water, 2.5 M CaCl_2_ was added (final concentration: 125 mM) and the solution was mixed dropwise with 2x HEPES-buffered solution [HBS] (42 mM HEPES, 15 mM D-glucose, 1.4 mM Na_2_HPO_4_, 10 mM KCl, 274 mM NaCl 274 mM, pH 7.1). This mixture was added dropwise to 1 ml cells either suspended in 10% FBS supplemented DMEM or on attached cells. The cells were plated on poly-L-lysine-coated plates, and the medium was replaced with fresh DMEM after 6-7 hours.

### BRET Measurement

We performed the BRET experiments on adherent cells 24–28 hours after transfection using a Thermo Scientific Varioskan Flash multimode plate reader at 37°C as described previously (35). Briefly, we replaced the medium with modified Kreb’s-Ringer medium (120 mM NaCl, 10 mM glucose, 10 mM Na-HEPES, 4.7 mM KCl, 0.7 mM MgSO4, 1.2 mM CaCl_2_, pH 7.4). We determined the expression of the YFP- or Venus-tagged proteins by recording fluorescence intensity at 535 nm with excitation at 510 nm. After the addition of the luciferase substrate coelenterazine *h* (5 μm), we measured luminescence intensity every 85 seconds for 36–74 min at 530 nm and 480 nm using filters. The BRET ratio was determined by dividing the luminescence intensities with each other (I_530nm_/I_480nm_). To calculate the stimulus-induced BRET ratio change, we performed baseline BRET signal correction and subtracted the BRET ratios of the vehicle-treated cells from that of stimulated cells. All BRET measurements were performed at least in triplicate.

### Confocal Microscopy

To obtain confocal images of the cellular distribution of β-arrestin2 in living cells, HEK 293T cells were plated on poly-L-lysine-coated glass coverslips. The next day the cells were transfected with plasmids encoding unlabeled CB_2_ receptors and fluorescently labeled β-arrestin2. 24 h after transfection, the cells were stimulated with 10 μM JWH-133. After 1 hour, the medium was changed to modified Kreb’s-Ringer medium, and the localization of the probes was examined in living cells at 37°C with Zeiss LSM 710 confocal laser-scanning microscope using a ×63 objective.

To explore the intracellular localization of receptors, we transfected the cells on 6-well plates with the wild-type or the mutant receptors labeled with YFP (2 μg/well). To label the cell membranes and make the recognition of the cell edges easier, plasma membrane-targeted Cerulean (L10-Cerulean, Cerulean fused to the targeting sequence of Lyn kinase (40) was coexpressed in these cells (0.2 μg/well). Experiments were performed 48 hours after transfection. Cells were detached with trypsin and plated on 8 well Ibidi plates with 50.000 cells/well density. 4-5 hours later, cells were stimulated with JWH-133 (10 μM) for one hour, after which they were fixed in 4% paraformaldehyde for 10 minutes. 5x5 images were taken with 40x objectives. The cells were identified on the composite images using the cellpose cellular segmentation algorithm (41), https://github.com/MouseLand/cellpose) in ml-workspace docker environment (https://github.com/ml-tooling/ml-workspace). In the next step, the masks were applied to the YFP images to separate the cells. Using the scikit-image python library, the original masks were both dilated and eroded in 20 and 40 cycles, respectively, with one pixel at a time. Differences between two masks in consecutive steps gave concentric contours whose points defined a specific distance from the cell edge. Mean fluorescence was measured under these contour masks. Contours between dilation cycles 10 and 20 (most distant contours) were taken as background for each cell and were subtracted from all contour mean values. Contour at the cell edge was labeled with 0, intracellular contours with positive, extracellular values with negative indices. Membrane-to-cytoplasm ratios were calculated as the ratio of fluorescence under contours between 0 - 5 (membrane), and contours >5 (cytoplasm).

### Affinity Purification

HEK 293T cells were transfected in suspension with plasmids encoding wild-type or mutant CB_2_R–BirA (promiscuous biotin ligase, 0.5 μg/well) and β-arrestin2–Venus (0.125 μg/well) in 24-well plates. 24 h after transfection, cells were stimulated with 10 μM JWH-133 (CB_2_R agonist), and simultaneously 100 μM biotin was added for 20–24 h to allow substantial biotinylation of β-arrestin2–Venus. Reactions were stopped by placing the dishes on ice and washing with them ice-cold PBS solution. The washing step was repeated 3 times. Then the cells were lysed with RIPA buffer (50 mM Tris-HCl, 150 mM NaCl, 1% Triton X-100, 0.1% SDS, 0.25% sodium deoxycholate, 1 mM EDTA; pH 7.4) supplemented with cOmplete Protease Inhibitor mixture (Roche) and Phosphatase Inhibitor Mixture 3 (Sigma). Lysates were collected, rotated for 10 min at low speed, then centrifuged at 20,800 × *g* for 10 min. Supernatants were incubated with 30 μl of High Capacity NeutrAvidin-agarose resin (Thermo Scientific) for 20 h at 4°C, then the beads were washed 2 times for 30 minutes with ice-cold high salt RIPA (50 mM Tris-HCl, 900 mM NaCl, 1% Triton X-100, 0.1% SDS, 0.25% sodium deoxycholate, 250 mM LiCl, 1 mM EDTA; pH 7.4) and once with PBS. The beads were resuspended in PBS. YFP and fluorescence intensities were determined by exciting at 510 nm and measuring emission at 535, respectively, using a Thermo Scientific Varioskan Flash multimode plate reader.

### Immunoblot Analysis of GRKs

HEK 293T cells plated on 10 cm plates expressing wild-type or mutant CB_2_R–BirA (10 μg/well) were treated with JWH133 and biotin similarly as above described. Proteins were eluted from HEK 293T cell extracts in SDS lysis buffer containing biotin and 10% mercaptoethanol. The samples were boiled and centrifuged. Proteins were separated with SDS-polyacrylamide gel electrophoresis and were blotted onto PVDF membranes. Membranes were treated with antibodies against GRK2 (C-15) or GRK3 (C-14) (sc-562 and sc-563, Santa Cruz) followed by the treatment with HRP-conjugated secondary antibodies. Blots were also stained with Alexa680-streptavidin (ThermoFisher) to assess the total protein amounts in the pull-downs. Visualization was made with Immobilon Western chemiluminescent HRP Substrate (Millipore), and fluorescence was detected with Azure c600 (Biosystems). The results were quantitatively evaluated with densitometry (ImageJ).

### CB_2_R Structure Depiction and Molecular Dynamics

We used a refined CB_2_R structure with bound CP55,940 published recently (42). For molecular modeling, molecular dynamics, and analysis the YASARA tool was used (43). The original receptor structure was subjected to *in silico* “point mutations” resulting in the L133I and Q63R structure variants. The ‘runmembrane’ macro of the supplied macro library was applied for the three structures, that is: immersion of the receptor to a membrane; balancing the charges; hydration of the system; applying periodic box conditions; initial energy minimization by steepest descent and then simulated annealing method; MD simulation on 298 K° with electrostatic interactions up 8 Å, simulation snapshots were taken at 250 ps intervals. Reference molecule structures have been sampled from the MD simulation frames and depicted using UCSF Chimera 1.14 software (44).

### GloSensor Assay

HEK 293T cells were transfected with or without an untagged CB_2_R construct (0.2 μg/well) and GloSensor™ (Promega, 2μg/well) plasmid, and were plated on 6-well plates. The next day cells were detached with trypsin and plated on 96 well plates with 50.000-100.000 cells/well density. Experiments were performed 48 hours after transfection. Before the measurement the medium of the cells was changed to colorless HBSS buffer (Hank’s Balanced Salt Solution: 1.25 mM CaCl_2_.2H_2_O, 0.5 mM MgCl_2_.6H_2_O, 0.4 mM MgSO_4_.7H_2_O, 5.4 mM KCl, 0.4 mM KH_2_PO_4_, 4.2 mM NaHCO_3_, 137 mM NaCl, 0.3 mM Na_2_HPO_4_, 5.5 mM D-glucose pH 7.4) containing 1 mM luciferin and 0.1% BSA. To load the cells with luciferin, the plates were incubated at room temperature for 2 hours, protected from light. After the incubation period, bioluminescence was recorded using a VarioSkan Flash plate reader (0.3 s/well) at 37°C. The cells were treated with cannabinoid agonist ligands (JWH-133 and 2-AG) in increasing concentrations. Cells were incubated with the agonists for 4 min, and then the cAMP signal was induced by the stimulation of the endogenous β_2_-adrenergic receptors with 1 μM isoproterenol (ISO). The inhibition of the ISO-induced cAMP production was analyzed by comparing the bioluminescence intensities at 7 minutes after the addition of ISO.

### Statistical Analysis

Data are presented as mean ± standard error of the mean (S.E.M). GraphPad Prism 9.0.0. software or python matplotlib and seaborn libraries were used for graph construction, statistical analysis, and curve fitting. The results were analyzed by two-way ANOVA and Tukey’s post-hoc test was applied for pairwise comparisons of the wild-type and mutant CB_2_Rs.

## Results

### CB_2_R Variants Have Similar G Protein Activation in HEK293T Cells

**Figure 1:**
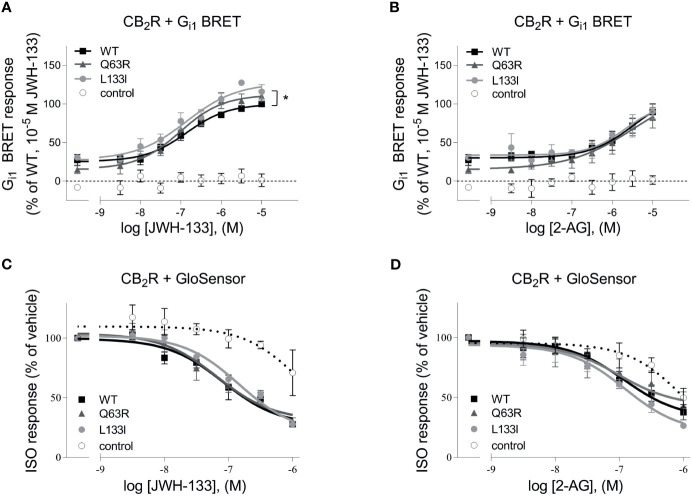
**(A, B)** G protein activity followed by BRET: HEK 293T cells were transfected with the indicated CB_2_R, Gαi1–Rluc8, Venus–β1, and γ2 DNA constructs. Concentration-response curves showing G protein activation of CB_2_ receptors: CB_2_R-WT (black squares) CB_2_R-Q63R (grey triangles) and CB_2_R-L133I (grey circles) in HEK 293T cells under basal and JWH-133 **(A)** or 2-AG-stimulated **(B)** conditions. The results were analyzed by two-way ANOVA (stimulation and expressed receptor) and Tukey’s post-hoc test was applied for pairwise comparisons of the wild-type and mutant CB_2_R. * indicates a significant difference between wild-type CB_2_R *vs.* CB_2_R-L133I (p<0.01). No other comparison between receptors was significantly different, but all receptors differed from control (p<0.001). The mean ± S.E.M. of the data from 4 independent experiments is shown. **(C, D)** cAMP signaling: ISO-induced cAMP signal decreases with CB_2_R stimulation. Cells were co-transfected with CB_2_Rs and GloSensor, and no CB_2_R was expressed in control cells. The cAMP signal was induced with 1 μM ISO. Data show the effect of JWH-133 **(C)** or 2-AG **(D)** treatment on cAMP formation by the three CB_2_ receptors. The results were analyzed with two-way ANOVA (stimulus and expressed receptor) and Tukey’s post-hoc test was applied for pairwise comparisons of the wild-type and mutant CB_2_R. There was no significant difference between the CB_2_Rs.

First, we tested whether the two studied CB_2_R polymorphisms, CB_2_R-Q63R and CB_2_R-L133I, affect the G protein activation of the CB_2_R (**Figure 1**). CB2R is known to activate the G_i/o_ subfamily of G proteins and decrease the intracellular cAMP levels. We assessed the basal and the agonist-induced G_i1_ activation of untagged CB_2_Rs using a G_i1_ bioluminescence resonance energy transfer (BRET) activation sensor. The basal activity was determined by treatment with the inverse agonist AM630, and agonist-induced activation in the first 30 minutes was analyzed using 2-arachidonoylglycerol (2-AG) and JWH-133 as agonists. We found no significant difference between the agonist-induced concentration-response curves of the wild-type and the CB_2_R-Q63R receptors (**Figures 1A, B**). Although the basal activity in the case of the CB_2_R-Q63R was slightly lower, the difference was not significant. On the other hand, when stimulated with JWH133, but not with 2-AG, the efficacy in the case of CB_2_R-L133I was enhanced compared to CB_2_R-WT. We also tested the effect of CB_2_R mutants on cAMP level, the downstream signaling event of the G_i/o_ proteins. We measured the changes in the cAMP signal induced by the stimulation of endogenous β_2_-adrenergic receptors using a luciferase-based cAMP probe, GloSensor (**Figures 1C, D**). The cAMP signal was inhibited by the simultaneous activation of the G_i/o_-activating CB_2_Rs, and no significant difference was detected between the wild-type and the mutant CB_2_Rs upon activation.

### CB_2_R Variants Have Distinct β-Arrestin2 and GRK Binding Properties

**Figure 2:**
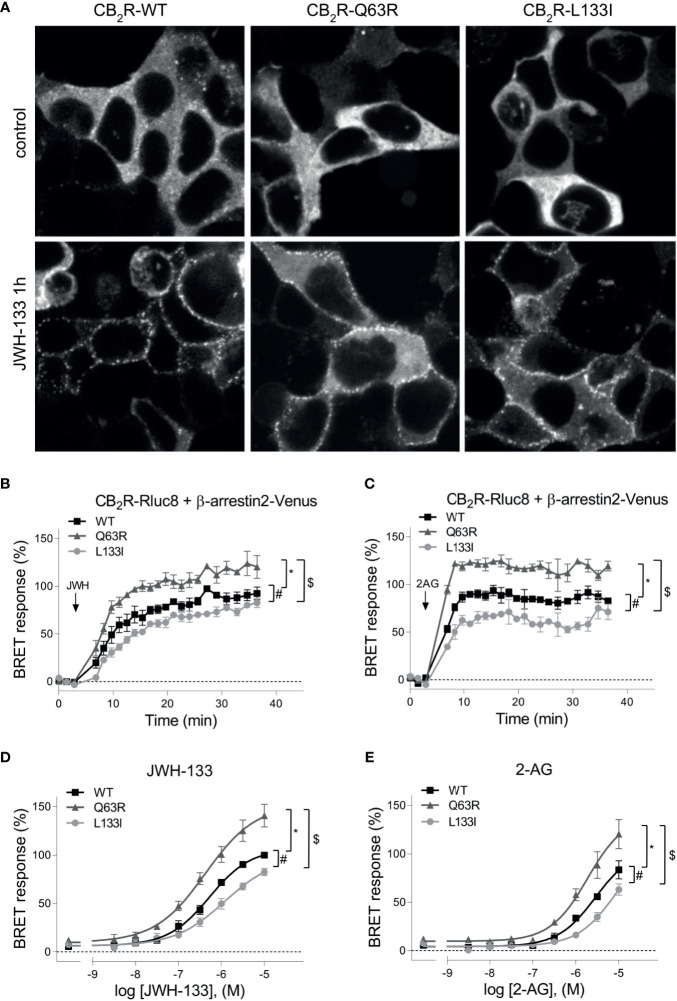
**(A)** β-arrestin2 localization: HEK 293T cells were co-transfected with unlabeled CB_2_Rs and β-arrestin2–Venus. Cells were untreated (control) or stimulated with JWH-133 (10 μM), for 1 hour. The cells were visualized by laser scanning confocal microscopy. **(B–E)** β-arrestin2 coupling to CB_2_Rs: BRET measurements showing the recruitment of β-arrestin2 to CB_2_ receptors upon agonist stimulus. CB_2_R-Rluc8 constructs (CB_2_R-WT: black squares, CB_2_R-Q63R: grey triangles, or CB_2_R-L133I: grey circles) were co-expressed with β-arr–Venus in HEK 293T cells, and BRET was measured upon JWH-133 (10 μM, **B**) or 2-AG (10 μM, **C**) stimulus. Data are shown as the percentage of the maximal response to 10^-5^ M JWH-133. Measurements were baseline-corrected to vehicle data (indicated by horizontal dashed line). Arrows indicate the time point of stimulation. **(C)** Concentration-response curves showing the recruitment of β-arrestin2 to CB_2_ receptors: in HEK 293T cells under basal and different JWH-133 (logEC50: -6.276; -6.373; -5.922 for wild-type, Q63R and L133I CB2 receptors) **(D)** or 2-AG-stimulated conditions (logEC50: -5.538; -5.725; -5.040 for wild-type, Q63R and L133I CB2 receptors) **(E)**. The results were analyzed by two-way ANOVA (stimulation and mutation) and Tukey’s post-hoc test was applied for pairwise comparisons of the wild-type and mutant CB_2_R. The mean ± S.E.M. of the data in the form of 4 experiments is in the results. *, #, $ indicate a significant difference between control *vs.* CB_2_R-Q63R, control *vs.* CB_2_R-L133I, and CB_2_R-L133I *vs*. CB_2_R-Q63R, respectively (p<0.001).

In addition to the G protein activation, another important event following GPCR activation is the binding of β-arrestins. Therefore, we next examined the ability of the mutant receptors to bind these proteins. CB_2_R binds β-arrestins transiently at the vicinity of the plasma membrane suggesting that it is a class A receptor (45, 46). Since CB_2_R, similarly to other class A receptors, is known to bind β-arrestin2 stronger than β-arrestin1 (47), we focused on β-arrestin2. First, we followed β-arrestin2 recruitment with confocal microscopy (**Figure 2A**). Agonist stimulation of all three receptors resulted in plasma membrane translocation of Venus-tagged β-arrestin2, whereas no β-arrestin2 on intracellular vesicles was observed. This confirms the transient nature of the coupling of these two proteins. Visually no significant difference was observed between the receptor subtypes, so to quantitatively analyze the extent of β-arrestin2 binding, we performed real-time bioluminescence resonance energy transfer (BRET) measurements. In these experiments, BRET signal was detected between RLuc8-tagged CB_2_Rs and Venus-tagged β-arrestin2 (**Figures 2B, E**). Interestingly, CB_2_R-Q63R mutant had increased, whereas CB_2_R-L133I had decreased β-arrestin2 binding compared to the CB_2_R-WT upon both JWH-133 and 2-AG stimuli.

**Figure 3:**
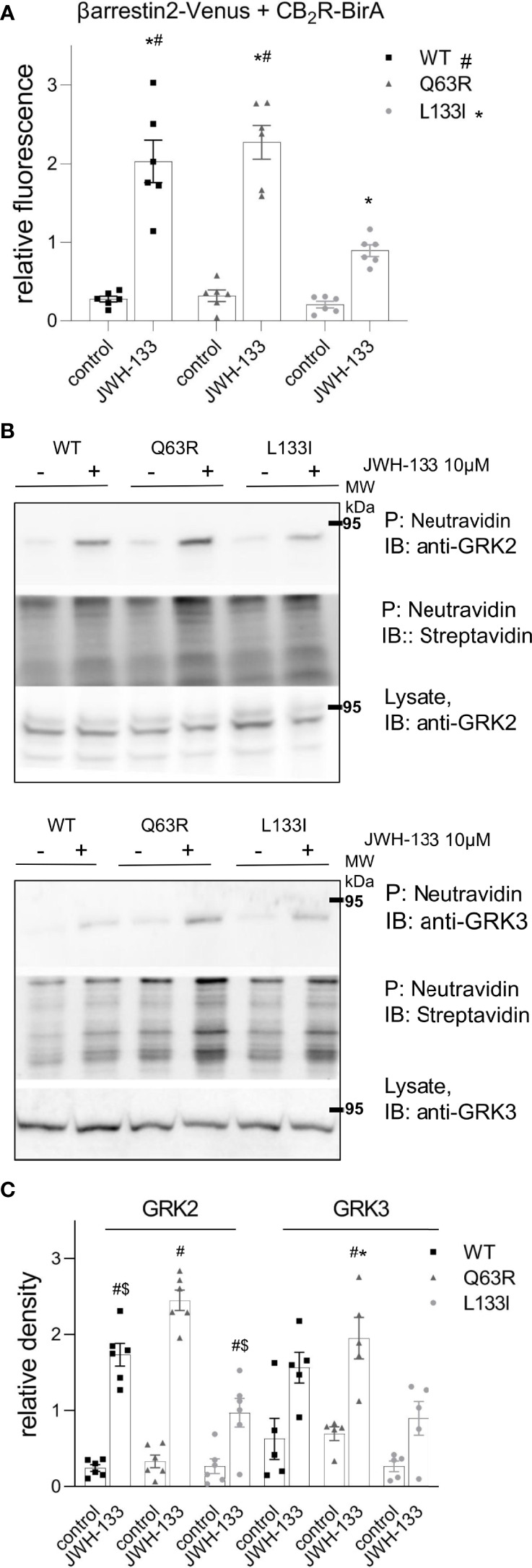
Proximity biotinylation assays: HEK 293T cells were either co-transfected with plasmids encoding wild type or mutant CB_2_R–BirA and β-arrestin2–Venus **(A)** or transfected only with BirA-tagged CB_2_Rs **(B, C)**. 24 h after the transfection, cells were stimulated with 10 μM JWH-133, and at the same time 100 μM biotin was added for 20–24 h **(A)** The cells were lysed and the biotinylated β-arrestin2–Venus was pulled down using NeutrAvidin beads. Total Venus fluorescence on the beads is shown ± S.E.M. Two-way ANOVA indicated significant effects on the variation of both the mutations and the stimulation (stimulation: p<0.001, mutation: p<0.01) **(B–D)** Interaction of CB_2_Rs with endogenous GRK2 and GRK3. Representative blots of pull-downs are shown on panel **(B)**. Quantified densities were normalized to streptavidin staining and data are shown as individual samples and mean ± S.E.M. (n=6-5) **(C)**. The results were analyzed by two-way ANOVA (stimulation and mutation) and Tukey’s post-hoc test was applied for pairwise comparisons of the wild-type and mutant CB_2_R. For both GRKs, two-way ANOVA indicated significant effects on the variation of both the mutations and the stimulation (stimulation: p<0.01 for both, mutation: p<0.001 for GRK2 and p<0.05 for GRK3, respectively). # indicates significant difference *vs.* mock-stimulated samples, * indicates a significant difference compared to L133I mutant and $ indicates a significant difference compared to Q63R in pairwise comparisons in the post-hoc test (p<0.05) **(A, C)**.

To verify the results above in another experimental setup, we used proximity biotin-labeling and quantified the interaction between CB_2_Rs and β-arrestin2. HEK 293T cells were co-transfected with receptors labeled with BirA-R188G biotin ligase (CB_2_-BirA) and β-arrestin2–Venus. R188G mutation turns BirA into a promiscuous biotin ligase, which biotinylates all proteins in the vicinity of the BirA-R118G-labeled protein (37). Interaction between CB_2_R-BirA and β-arrestin2–Venus was induced by stimulation with 10 μM JWH-133, and the biotinylated proteins were pulled down with NeutrAvidin beads. The fluorescence of β-arrestin2–Venus bound to the beads was then measured. JWH-133 induced β-arrestin2 binding both to the wild-type and the mutant CB_2_-BirA receptors. CB_2_R-Q63R-BirA stimulation led to a slightly elevated, whereas CB_2_R-L133I-BirA stimulation led to a decreased β-arrestin2–Venus signal, compared to the wild-type receptor (**Figure 3A**).

β-arrestin binding to GPCRs is regulated by GRK kinases. To test whether mutations in CB_2_R affect GRK recruitment, we performed further proximity biotinylation experiments. After stimulation of the receptors, biotinylated endogenous proteins were pulled down and GRK2 and GRK3 were detected with immunoblotting (**Figure 3B**). The results show that upon stimulation of the receptors with JWH-133, endogenous GRK2 and GRK2 were enriched in samples, showing their interaction with the activated receptor. Interestingly, the GRK binding pattern to CB_2_Rs correlated with the binding of β-arrestin2 (**Figures 3B, C** **)**. These results suggest that GRK-binding preference to the receptor may contribute to the observed differences in β-arrestin2 binding (**Figure 3B, C** **)**.

### CB_2_ Variants Have Altered Intracellular Trafficking

**Figure 4:**
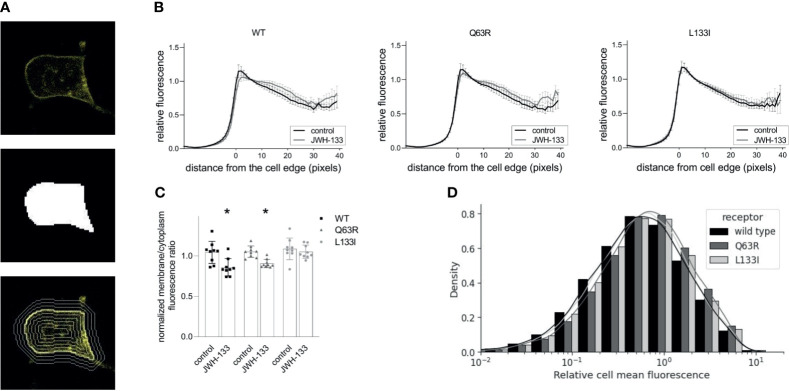
CB2R distribution in HEK 293T cells following stimulation with JWH-133: HEK 293T cells were co-transfected with YFP-tagged CB_2_R isoforms and L10-cerulean. Confocal microscopy images were taken with Zeiss LSM 710 confocal laser-scanning microscope, and the cells were detected with cellpose, a neural network-based algorithm. For each cell, a mask covering the cell was determined (**A**, top, and middle), and the mask was iteratively dilated or eroded resulting in a total of 60 contours (examples are shown on **A**, bottom). **(B)** Cell fluorescence profile was determined by measuring average fluorescence under each contour in control and stimulated HEK293T cells. Cells expressed either CB_2_R-WT-YFP, CB_2_R-Q63R-YFP, or CB_2_R-L133I-YFP. Mean values ± S.E.M. @ are shown (n=9, ~50000 cells total). **(C)** Membrane (0-5 pixels from cell edge)/cytoplasm (>5 pixels from cell edge) fluorescence ratios for the wild-type and mutant CB_2_Rs in control and stimulated cells. Mean ± S.E.M. @ are shown from n=9 experiments. * indicates a significant difference between control and stimulated samples (p<0.05), analyzed with two-way ANOVA (stimulation, mutation) using Tukey’s *post hoc* test for multiple comparisons. **(D)** Cell fluorescence distribution in 9 experiments representing ~50000 cells. Fluorescence is normalized to average overall cell fluorescence in each experiment.

To assess the intracellular distribution of the mutant receptors, we expressed yellow fluorescent protein (YFP)-tagged CB_2_Rs in HEK 293T cells. After taking confocal microscopy images, we identified the cells using the cellpose cellular segmentation algorithm (41). We analyzed total fluorescence and the fluorescence intensity distribution of the receptors relative to the cell edge (**Figure 4A**). The receptors (wild-type, Q63R, and L133I) had similar expressions (**Figure 4D**) and cellular distributions (**Figures 4B, C**), with intensity peaks at the vicinity of the cell edge. The similar membrane expression of the receptors in cells suggests that the differences seen in β-arrestin2 binding are not caused by altered intracellular distributions. When the cells were stimulated with JWH-133 for 1 hour, the distribution profile changed considerably with lower fluorescence in the cell membrane and higher fluorescence in the cytoplasm for both wild-type and Q63R mutant receptors. However, in the case of the L133I mutation, the change in distribution was not significant (**Figures 4C, D**).

**Figure 5:**
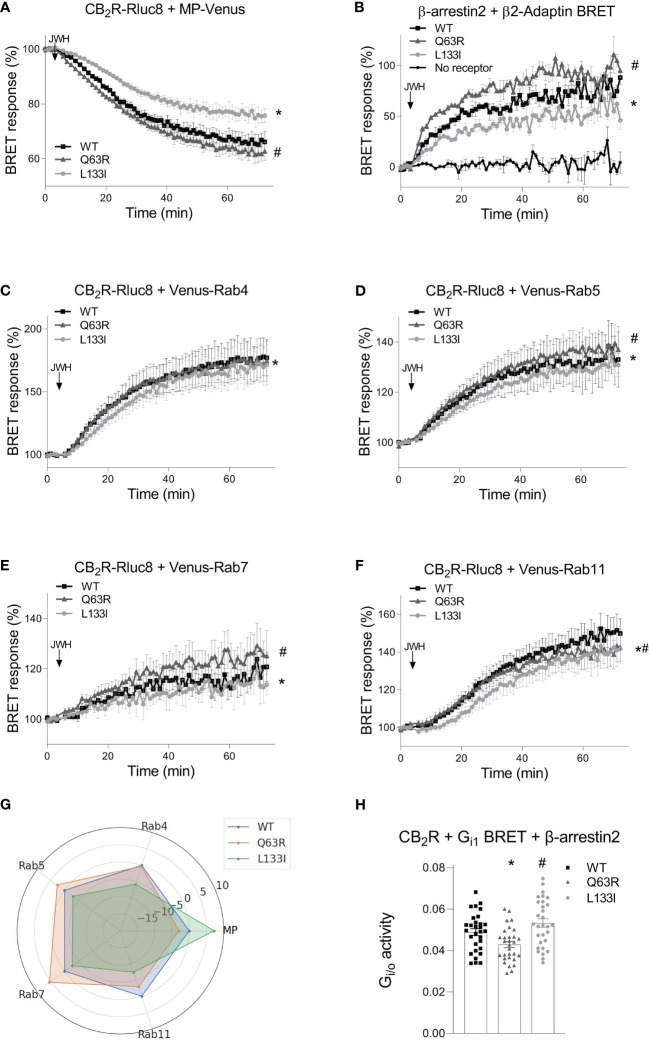
CB2R intracellular trafficking and β-arrestin2 translocation followed by bystander BRET: CB_2_R-Rluc8-WT (black squares) CB_2_R-Rluc8-Q63R (grey triangles) and CB_2_R-Rluc8-L133I (grey circles) were co-expressed with Venus-tagged membrane markers. The cell membrane was labeled with MP-Venus **(A)**, rapid recycling endosomes with Venus-Rab4 **(C)**, early endosomes with Venus-Rab5 **(D)**, late endosomes with Venus-Rab7 **(E)**, and late recycling endosomes with Venus-Rab11 **(F)**. β-arrestin2-Rluc8 was coexpressed with a clathrin-coated pit marker, β2-Adaptin-Venus **(B)**. The arrows show the time of the JWH-133 (10 µM) treatment. Statistical analysis was performed with two-way ANOVA (time, mutation) followed by Tukey’s post-hoc test with multiple comparisons. * and # indicate significant differences in pairwise comparisons, CB_2_R-L133I *vs.* CB_2_R-WT and CB_2_R-Q63R *vs.* CB_2_R-WT respectively (p<0.001). **(G)** Radial plot showing average differences compared to CB_2_R-WT across all timepoints. **(H)** HEK 293T cells were transfected with the indicated CB2R, Gαi1–Rluc8, Venus–β1, γ2 and β-arrestin2 DNA constructs. Columns show G protein activation of the CB2 receptors: CB2R-WT (black squares) CB2R-Q63R (grey triangles) and CB2R-L133I (grey circles) in HEK 293T cells after 2 hour JWH-133 stimulation (10 μM). The mean of the negative ΔBRET (G protein activity) values from 5 independent experiments were compared by one-way ANOVA (repeated measures) and Tukey’s post-hoc test was applied for pairwise comparisons of the wild-type and mutant CB2R. *# indicates a significant difference between wild-type CB2R *vs.* CB2R-Q63R and CB2R-L133I *vs.* CB2R-Q63R (p<0.01). All technical replicates from the 5 independent experiments are shown.

To further characterize the receptor trafficking with higher sensitivity, we followed the receptor disappearance from the cell membrane and their appearance in intracellular vesicles in bystander BRET experiments (**Figure 5**) (35, 39). BRET was detected between Rluc8-tagged receptors and a Venus-labeled either plasma membrane- or intracellular vesicle-localized marker. The plasma membrane was labeled with myristoylated-palmitoylated Venus (MP-Venus), whereas the intracellular vesicles were marked with different Rab small proteins also tagged with Venus fluorescent protein (Venus–Rab4 for rapid recycling endosomes, Venus–Rab5 for early endosomes, Venus–Rab7 for late endosomes and Venus–Rab11 for late recycling endosomes). We also followed the interaction of β-arrestin2–Rluc8 with β2-adaptin–Venus. β2-adaptin is a key protein in the initiation of clathrin-dependent endocytosis (48) (**Figure 5B**). An increase or decrease of the BRET signal indicates the appearance or disappearance of the CB_2_R at a specific cellular location, respectively. As shown in **Figure 5**, stimulation is followed by receptor disappearance from the membrane and appearance in intracellular vesicles. In parallel with the internalization of the receptors, β-arrestin2 also interacted with β2-adaptin (**Figures 5A, B** **)**. The internalization pattern corresponded to the β-arrestin2 binding patterns observed with the mutant receptors. Namely, CB_2_R-Q63R, which has stronger coupling to β-arrestin2, also had slightly enhanced disappearance from the membrane and appearance in Rab5 and Rab11 endosomes. CB_2_R-L133I, which had weaker coupling to β-arrestin2, showed slower internalization and arrival into all four types of endosomes (**Figure 5A–G**). Since prolonged stimulation resulted in distinct intracellular distribution of the mutant receptors, we retested the G protein activity after 2 hours of continuous stimulation with JWH-133 with Gi1 BRET sensor in cells also overexpressing β-arrestin2. In this setup, the L133I mutant had increased G protein response, while the Q63R showed decreased G protein activation compared to the wild-type receptor. This result correlates with the differences observed in the degree of internalization and cellular distribution of the mutant receptors (**Figure 5H**).

### Isoleucine at Position 133 Alters the ICL2’s Protrusion Towards the Cytoplasm

**Figure 6:**
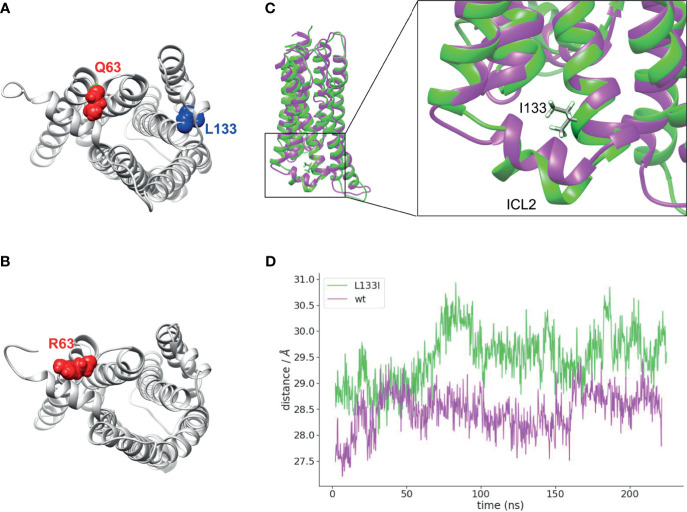
Localizations of Q63 and L133 amino acids and molecular dynamics simulation: **(A)** Q63 (red) amino acid is located on the cytoplasmic surface of the CB_2_R, whereas L133 (blue) is located on the outer side of the TM3. CB_2_R receptor structure is shown from the cytoplasmic side. **(B)** R63 amino acid position on the CB_2_R cytoplasmic site. **(C)** Superposed structures of CB_2_R-WT (magenta) and CB_2_R-L133I (green). ILC2 is pushed towards the cytoplasm in the CB_2_R-L133I. **(D)** Distances are shown between the centers of masses of the TM helices and the ICL2 amino acids during the simulation.

To gain an insight into the structural changes induced by the two CB_2_R mutants, we carried out molecular dynamics simulations with CB2R bound to cannabinoid receptor agonist, CP55,940 (42) on the wild-type, Q63R, and L133I receptors embedded into a lipid bilayer. In the case of the CB_2_R-Q63R, no major structural rearrangements have been observed (not shown), although arginine is sterically larger compared to the glutamine, and results in an increased number of positive charges on the cytoplasmic side of the receptor (**Figures 6A, B** **)**. On the other hand, isoleucine in position 133 is positioned on the outer side of the third helix (**Figures 6A, C** **)**, and its γ_2_ carbon atom’s position results in a movement of the second intracellular loop towards the cytoplasm (**Figures 6C, D** **)**.

## Discussion

In this study, we examined two missense polymorphisms of the CB_2_R, which may contribute to the development of a variety of human diseases. We explored the effects of the mutations on the G protein activation, β-arrestin2 binding, intracellular distribution, and trafficking. We detected the most striking changes in their β-arrestin2 binding properties, namely CB_2_R-Q63R had increased, whereas CB_2_R-L133I had decreased coupling compared to the wild-type receptor. The alteration of their ability to activate G proteins, on the other hand, was less pronounced, only CB_2_R-L133I showed some enhanced activation when JWH-133 agonist in the G_i1_ protein BRET experiments was assessed. Although one could expect stronger G protein activation in case of weaker β-arrestin binding and desensitization, we could detect differences neither with the endogenous agonist, 2-AG, nor when the endogenous cAMP levels were assessed. This suggests that even if the G protein activation is altered, the differences are minimal. Our results in the case of the CB_2_R-Q63R’s G protein activation are in contrast to those previously reported (49). In the study of Carrasquer et al., G protein activation was weaker in the receptor carrying the R mutation. Although the reason for the difference is not clear, there are methodological differences between the two studies. They measured the cAMP signals induced by forskolin in the presence of phosphodiesterase inhibitors, which might result in increased sensitivity in their assays. Also, the G protein assays might be sensitive to receptor expression differences. However, when we stimulated the receptors for a prolonged time, their G protein activations correlated with the intracellular redistribution of the receptors, CB2R-L133I having stronger and CB2R-Q63R having weaker G protein activity. Nevertheless, the decreased cAMP signal would be in good agreement with the increased β-arrestin2-binding of this mutant. Similarly, decreased Erk1/2 activation by the CB_2_R-Q63R might also be the consequence of the enhanced desensitization by the arrestins (50).

To assess β-arrestin2 coupling to CB_2_R, we used both BRET measurements and a proximity-labeling technique with BirA-labeled receptors. BRET experiments showed enhanced binding to CB_2_R-Q63R and decreased coupling to CB_2_R-L133I. Although with the proximity biotinylation method only the effect of the L133I mutation was significant, it has to be noted that in proximity biotinylation experiments the cells have been stimulated for ~18 hours, which might lead to biotinylation of β-arrestins in multiple coupling-uncoupling cycles, eventually until all the expressed β-arrestins are labeled. Thus, the method might not be sensitive enough for differentiating modest differences, especially if the binding is already sufficiently strong.

When receptor-β-arrestin binding experiments are evaluated, the membrane expression of the receptors has to be also addressed. Since CB_2_R binds β-arrestin only near to the cell membrane, higher or lower receptor membrane expressions themselves may lead to bigger or lower β-arrestin2 BRET signals, respectively. We assessed the intracellular distributions of the CB_2_Rs in confocal images using computer-aided high-throughput analysis. The applied cellpose algorithm enables the separation of the cells in microscopic images, and the analysis can be carried out on each cell separately. We analyzed the spatial fluorescence profile of the cells. The analysis showed that the distribution, as well as the total fluorescence of the three CB_2_Rs, are not significantly different. Thus, the differences in β-arrestin2 binding of the two mutant CB_2_Rs cannot be explained by localization and expression differences, but on the contrary, altered β-arrestin2 binding may result in the changes observed in the ligand-induced internalization and appearances in the late endosomes and recycling. Namely, in the case of the CB_2_R-Q63R, stronger β-arrestin2-coupling seems to result in enhanced internalization and trafficking to Rab5 and late Rab7 endosomes, whereas weaker β-arrestin2-binding of CB_2_R-L133I leads to a slower rate of internalization and weaker appearance in all intracellular vesicles (**Figure 5G**). Thus, changes in β-arrestin2 binding of the CB_2_Rs affect their intracellular trafficking, which in turn may lead to altered signaling and may offer an explanation for the observed clinical consequences. Neither of the two mutations affects the serine/threonine amino acids in the C-terminal tail of the receptor since they reside on the first (Q63R) or near the third (L133I) intracellular loops. There are at least two possible explanations for the differences seen in β-arrestin binding between the wild-type receptor and the two mutants. First, the mutations might affect GRK binding to the receptor and have an effect on the receptor phosphorylation. Indeed, GRK2 binding correlated well with the β-arrestin2 binding pattern of the two mutations. Secondly, mutations in Q63 and L133 amino acids might affect the binding of the β-arrestin2 directly. β-arrestin-GPCR interactions are composed of at least two interaction sites: the interaction with the C-terminus and the core interaction. The core interaction involves the protrusion of the finger loop into the transducer pocket of the GPCRs and several other interactions between the second and the third intracellular loops (ICL2 and ICL3, respectively) (51). Q63 resides in the ICL2, and the replacement of this amino acid to arginine brings an increased number of positive charges to the receptor-β-arrestin2 interface, possibly changing the binding properties of these two proteins. In the case of the L133I mutation, the possible effect is not that obvious. The amino acid resides in the third helix of the receptor, with its side chain pointing towards the outer side of the receptor, and is not likely to be directly involved in the receptor-β-arrestin2 interaction. The leucine-isoleucine change also does not warrant major structural or charge changes. Therefore, we carried out molecular dynamics simulations using a recently described CB_2_R model in which the receptor active state is stabilized with a high-affinity agonist, CP55,940 (**Figures 6C, D**). According to these simulations, the methyl group of the γ_2_ carbon atom in the isoleucine clashes with the amino acids 140-141 in ICL2 of the wild-type structure, forcing it towards the cytoplasm. This movement might interfere with the receptor-β-arrestin interaction, decreasing the affinity of the binding.

Although the differences in the receptor-β-arrestin2 binding between the wild-type and the mutant receptors are relatively small, these changes significantly affect the cellular distribution of the receptors after their prolonged stimulation. These differences may in turn lead to altered downstream signaling events, where the differences may be even more exaggerated due to the signal amplification steps. In further studies, it would be interesting to test the effect of endogenous or exogenous cannabinoids on the downstream signaling of cells that express CB2R endogenously, such as peripheral immune cells, microglia, and neuronal cells, derived from subjects harboring wild-type or variant CB2R. These investigations would further help understand the role of CB2R variants in the development of the reported immune and psychoneurological disorders.

In conclusion, we show that two commonly occurring CB_2_R missense mutations, Q63R and L133I mutations affect the receptor’s ability to bind β-arrestin2. Since the G protein activations seem to be very similar or might be even enhanced in the case of the L133I mutant, these changes lead to biased signaling of the CB_2_R and could explain the clinical observation linked to these mutations. Moreover, since biased CB_2_R agonists are being developed (21, 52, 53), pharmacological strategies targeting the β-arrestin-binding of the CB_2_R might be options for further research in diseases affected by these mutations.

## Data Availability Statement

The raw data supporting the conclusions of this article will be made available by the authors, without undue reservation.

## Author Contributions

Conception and design of the experiments was undertaken by GT, AT, AB, LH, and MC. The experiments were performed by GT, AT, CJ, and ES-K. Molecular dynamics simulation were made by MCs and GT. Analysis was carried out by GT, AT, ÁM, ES-K, and MCs. Manuscript was prepared by GT, AT, ES-K, MCs, AB, and LH. All authors contributed to the article and approved the submitted version.

## Funding

This research was funded by grants Marie Curie Actions International Outgoing Fellowships (IOF) FP7-PEOPLE-2009-IOF-253628, OTKA K-116954 and VEKOP-2.3.2-16-2016-00002.

## Conflict of Interest

The authors declare that the research was conducted in the absence of any commercial or financial relationships that could be construed as a potential conflict of interest.

## Publisher’s Note

All claims expressed in this article are solely those of the authors and do not necessarily represent those of their affiliated organizations, or those of the publisher, the editors and the reviewers. Any product that may be evaluated in this article, or claim that may be made by its manufacturer, is not guaranteed or endorsed by the publisher.

## References

[1] Console-Bram L Marcu J Abood ME. Cannabinoid Receptors: Nomenclature and Pharmacological Principles. Prog Neuropsychopharmacol Biol Psychiatry (2012) 38:4–15. 10.1016/j.pnpbp.2012.02.009 22421596 PMC3378782

[2] Howlett AC. The Cannabinoid Receptors. Prostaglandins Other Lipid Mediat (2002) 68-69:619–31. 10.1016/S0090-6980(02)00060-6 12432948

[3] Pulgar TGDEL del Pulgar TG Velasco G Guzmán M. The CB1 Cannabinoid Receptor Is Coupled to the Activation of Protein Kinase B/Akt. Biochem J (2000) 347:369. 10.1042/0264-6021:3470369 10749665 PMC1220968

[4] Kobayashi Y Arai S Waku K Sugiura T. Activation by 2-Arachidonoylglycerol, an Endogenous Cannabinoid Receptor Ligand, of P42/44 Mitogen-Activated Protein Kinase in HL-60 Cells. J Biochem (2001) 129:665–9. 10.1093/oxfordjournals.jbchem.a002904 11328586

[5] Munro S Thomas KL Abu-Shaar M. Molecular Characterization of a Peripheral Receptor for Cannabinoids. Nature (1993) 365:61–5. 10.1038/365061a0 7689702

[6] Aymerich MS Aso E Abellanas MA Tolon RM Ramos JA Ferrer I. Cannabinoid Pharmacology/Therapeutics in Chronic Degenerative Disorders Affecting the Central Nervous System. Biochem Pharmacol (2018) 157:67–84. 10.1016/j.bcp.2018.08.016 30121249

[7] Schneider U Muller-Vahl KR Stuhrmann M Gadzicki D Heller D Seifert J. The Importance of the Endogenous Cannabinoid System in Various Neuropsychiatric Disorders. Fortschr Neurol Psychiatr (2000) 68:433–8. 10.1055/s-2000-7734 11103679

[8] Ishiguro H Horiuchi Y Ishikawa M Koga M Imai K Suzuki Y. Brain Cannabinoid CB2 Receptor in Schizophrenia. Biol Psychiatry (2010) 67:974–82. 10.1016/j.biopsych.2009.09.024 19931854

[9] Onaivi ES Ishiguro H Gong JP Patel S Meozzi PA Myers L. Functional Expression of Brain Neuronal CB2 Cannabinoid Receptors Are Involved in the Effects of Drugs of Abuse and in Depression. Drug Addiction: Res Front Treat Adv (2008) 1139:434–49. 10.1196/annals.1432.036 PMC3922202 18991891

[10] Ishiguro H Iwasaki S Teasenfitz L Higuchi S Horiuchi Y Saito T. Involvement of Cannabinoid CB2 Receptor in Alcohol Preference in Mice and Alcoholism in Humans. Pharmacogenomics J (2007) 7:380–5. 10.1038/sj.tpj.6500431 17189959

[11] Chen X Zheng C Qian J Sutton SW Wang Z Lv J. Involvement of β-Arrestin-2 and Clathrin in Agonist-Mediated Internalization of the Human Cannabinoid CB2 Receptor. Curr Mol Pharmacol (2014) 7:67–80. 10.2174/1874467207666140714115824 25023974

[12] Shoemaker JL Ruckle MB Mayeux PR Prather PL. Agonist-Directed Trafficking of Response by Endocannabinoids Acting at CB2 Receptors. J Pharmacol Exp Ther (2005) 315:828–38. 10.1124/jpet.105.089474 16081674

[13] Atwood BK Wager-Miller J Haskins C Straiker A Mackie K. Functional Selectivity in CB2 Cannabinoid Receptor Signaling and Regulation: Implications for the Therapeutic Potential of CB2 Ligands. Mol Pharmacol (2012) 81:250–63. 10.1124/mol.111.074013 PMC3263955 22064678

[14] Reiter E Lefkowitz RJ. GRKs and β-Arrestins: Roles in Receptor Silencing, Trafficking and Signaling. Trends Endocrinol Metab (2006) 17:159–65. 10.1016/j.tem.2006.03.008 16595179

[15] Khoury E Nikolajev L Simaan M Namkung Y Laporte SA. Differential Regulation of Endosomal GPCR/β-Arrestin Complexes and Trafficking by MAPK. J Biol Chem (2014) 289:23302–17. 10.1074/jbc.M114.568147 PMC4156072 25016018

[16] Tohgo A Pierce KL Choy EW Lefkowitz RJ Luttrell LM. β-Arrestin Scaffolding of the ERK Cascade Enhances Cytosolic ERK Activity But Inhibits ERK-Mediated Transcription Following Angiotensin AT1a Receptor Stimulation. J Biol Chem (2002) 277:9429–36. 10.1074/jbc.M106457200 11777902

[17] Turu G Balla A Hunyady L. The Role of β-Arrestin Proteins in Organization of Signaling and Regulation of the AT1 Angiotensin Receptor. Front Endocrinol (2019) 10:519. 10.3389/fendo.2019.00519 PMC6691095 31447777

[18] Luttrell LM Lefkowitz RJ. The Role of Beta-Arrestins in the Termination and Transduction of G-Protein-Coupled Receptor Signals. J Cell Sci (2002) 115:455–65. 10.1242/jcs.115.3.455 11861753

[19] Peterson YK Luttrell LM. The Diverse Roles of Arrestin Scaffolds in G Protein–Coupled Receptor Signaling. Pharmacol Rev (2017) 69:256–97. 10.1124/pr.116.013367 PMC5482185 28626043

[20] Gurevich VV Gurevich EV. GPCR Signaling Regulation: The Role of GRKs and Arrestins. Front Pharmacol (2019) 10:125. 10.3389/fphar.2019.00125 30837883 PMC6389790

[21] Laprairie RB Bagher AM Denovan-Wright EM. Cannabinoid Receptor Ligand Bias: Implications in the Central Nervous System. Curr Opin Pharmacol (2017) 32:32–43. 10.1016/j.coph.2016.10.005 27835801

[22] McNeill SM Baltos J-A White PJ May LT. Biased Agonism at Adenosine Receptors. Cell Signal (2021) 82:109954. 10.1016/j.cellsig.2021.109954 33610717

[23] Ferraino KE Cora N Pollard CM Sizova A Maning J Lymperopoulos A. Adrenal Angiotensin II Type 1 Receptor Biased Signaling: The Case for “Biased” Inverse Agonism for Effective Aldosterone Suppression. Cell Signal (2021) 82:109967. 10.1016/j.cellsig.2021.109967 33640432

[24] Gurevich VV Gurevich EV. Biased GPCR Signaling: Possible Mechanisms and Inherent Limitations. Pharmacol Ther (2020) 211:107540. 10.1016/j.pharmthera.2020.107540 32201315 PMC7275904

[25] Oyagawa CRM de la Harpe SM Saroz Y Glass M Vernall AJ Grimsey NL. Cannabinoid Receptor 2 Signalling Bias Elicited by 2,4,6-Trisubstituted 1,3,5-Triazines. Front Pharmacol (2018) 9(1202):1–19. 10.3389/fphar.2018.01202 30524271 PMC6256112

[26] Dhopeshwarkar A Mackie K. Functional Selectivity of CB2 Cannabinoid Receptor Ligands at a Canonical and Noncanonical Pathway. J Pharmacol Exp Ther (2016) 358:342–51. 10.1124/jpet.116.232561 PMC4959096 27194477

[27] Mlost J Kostrzewa M Borczyk M Bryk M Chwastek J Korostyński M. CB2 Agonism Controls Pain and Subchondral Bone Degeneration Induced by Mono-Iodoacetate: Implications GPCR Functional Bias and Tolerance Development. Biomed Pharmacotherapy (2021) 136:111283. 10.1016/j.biopha.2021.111283 33482616

[28] Yates AD Achuthan P Akanni W Allen J Allen J Alvarez-Jarreta J. Ensembl 2020. Nucleic Acids Res (2019) 48:D682–8. 10.1186/s12864-021-07493-6 PMC7145704 31691826

[29] Tahamtan A Rezaiy S Samadizadeh S Moradi A Tabarraei A Javid N. Cannabinoid CB2 Receptor Functional Variation (Q63R) Is Associated With Multiple Sclerosis in Iranian Subjects. J Mol Neurosci (2019) 70(1):26–31. 10.1007/s12031-019-01395-9 31407233

[30] Ismail M Khawaja G. Study of Cannabinoid Receptor 2 Q63R Gene Polymorphism in Lebanese Patients With Rheumatoid Arthritis. Clin Rheumatol (2018) 37:2933–8. 10.1007/s10067-018-4217-9 30032418

[31] Bellini G Olivieri AN Grandone A Alessio M Gicchino MF Nobili B. Association Between Cannabinoid Receptor Type 2 Q63R Variant and Oligo/Polyarticular Juvenile Idiopathic Arthritis. Scand J Rheumatol (2015) 44:284–7. 10.3109/03009742.2015.1020863 25974389

[32] Rossi F Mancusi S Bellini G Roberti D Punzo F Vetrella S. CNR2 Functional Variant (Q63R) Influences Childhood Immune Thrombocytopenic Purpura. Haematologica (2011) 96:1883–5. 10.3324/haematol.2011.045732 PMC3232275 21828121

[33] Ezzat DA Hammam AA El-Malah WM Khattab RA Mangoud EM. Role of Cannabinoid CB2 Receptor Gene (CNR2) Polymorphism in Children With Immune Thrombocytopenic Purpura in Beni-Suef Governorate in Egypt. Egypt J Immunol (2017) 24:57–66. 29120578

[34] Minocci D Massei J Martino A Milianti M Piz L Di Bello D. Genetic Association Between Bipolar Disorder and 524A > C (Leu133Ile) Polymorphism of CNR2 Gene, Encoding for CB2 Cannabinoid Receptor. J Affect Disord (2011) 134:427–30. 10.1016/j.jad.2011.05.023 21658778

[35] Tóth AD Prokop S Gyombolai P Várnai P. Heterologous Phosphorylation–Induced Formation of a Stability Lock Permits Regulation of Inactive Receptors by β-Arrestins. J Biol (2018) 293(3):876–92. 10.1074/jbc.M117.813139 PMC5777260 29146594

[36] Gyombolai P Toth AD Timar D Turu G Hunyady L. Mutations in the “DRY” Motif of the CB1 Cannabinoid Receptor Result in Biased Receptor Variants. J Mol Endocrinol (2015) 54:75–89. 10.1530/JME-14-0219 25510402

[37] Roux KJ Kim DI Raida M Burke B. A Promiscuous Biotin Ligase Fusion Protein Identifies Proximal and Interacting Proteins in Mammalian Cells. J Cell Biol (2012) 196:801–10. 10.1083/jcb.201112098 PMC3308701 22412018

[38] Saulière A Bellot M Paris H Denis C Finana F Hansen JT. Deciphering Biased-Agonism Complexity Reveals a New Active AT1 Receptor Entity. Nat Chem Biol (2012) 8:622–30. 10.1038/nchembio.961 22634635

[39] Szakadáti G Tóth AD Oláh I Erdélyi LS Balla T Várnai P. Investigation of the Fate of Type I Angiotensin Receptor After Biased Activation. Mol Pharmacol (2015) 87:972–81. 10.1124/mol.114.097030 PMC4429721 25804845

[40] Várnai P Tóth B Tóth DJ Hunyady L Balla T. Visualization and Manipulation of Plasma Membrane-Endoplasmic Reticulum Contact Sites Indicates the Presence of Additional Molecular Components Within the STIM1-Orai1 Complex. J Biol Chem (2007) 282:29678–90. 10.1074/jbc.m704339200 17684017

[41] Stringer C Wang T Michaelos M Pachitariu M. Cellpose: A Generalist Algorithm for Cellular Segmentation. Nat Methods (2021) 18:100–6. 10.1038/s41592-020-01018-x 33318659

[42] Pandey P Roy KK Doerksen RJ. Negative Allosteric Modulators of Cannabinoid Receptor 2: Protein Modeling, Binding Site Identification and Molecular Dynamics Simulations in the Presence of an Orthosteric Agonist. J Biomol Struct Dyn (2020) 38:32–47. 10.1080/07391102.2019.1567384 30652534 PMC7487276

[43] Krieger E Vriend G. YASARA View—Molecular Graphics for All Devices—From Smartphones to Workstations. Bioinformatics (2014) 30:2981–2. 10.1093/bioinformatics/btu426 PMC4184264 24996895

[44] Pettersen EF Goddard TD Huang CC Couch GS Greenblatt DM Meng EC. and Analysis. J Comput Chem (2004) 25:1605–12. 10.1002/jcc.20084 15264254

[45] Oakley RH Laporte SA Holt JA Caron MG Barak LS. Differential Affinities of Visual Arrestin, Beta Arrestin1, and Beta Arrestin2 for G Protein-Coupled Receptors Delineate Two Major Classes of Receptors. J Biol Chem (2000) 275:17201–10. 10.1074/jbc.M910348199 10748214

[46] Reggio PH. Endocannabinoid Binding to the Cannabinoid Receptors: What is Known and What Remains Unknown. Curr Med Chem (2010) 17:1468–86. 10.2174/092986710790980005 PMC4120766 20166921

[47] Miljuš T Heydenreich FM Gazzi T Kimbara A Rogers-Evans M Nettekoven M. Diverse Chemotypes Drive Biased Signaling by Cannabinoid Receptors. bioRxiv (2020). 10.1101/2020.11.09.375162

[48] Hamdan FF Rochdi MD Breton B Fessart D Michaud DE Charest PG. Unraveling G Protein-Coupled Receptor Endocytosis Pathways Using Real-Time Monitoring of Agonist-Promoted Interaction Between Beta-Arrestins and AP-2. J Biol Chem (2007) 282:29089–100. 10.1074/jbc.M700577200 17675294

[49] Carrasquer A Nebane NM Williams WM Song ZH. Functional Consequences of Nonsynonymous Single Nucleotide Polymorphisms in the CB2 Cannabinoid Receptor. Pharmacogenet Genomics (2010) 20:157–66. 10.1097/FPC.0b013e3283367c6b 20124950

[50] Wang J Xu J Liu J Zhu H Peng Y Ding ZM. Genetic Variant Q63R of Cannabinoid Receptor 2 Causes Differential ERK Phosphorylation in Human Immune Cells. Genet Test Mol Biomarkers (2018) 22:320–6. 10.1089/gtmb.2018.0005 29694791

[51] Seyedabadi M Gharghabi M Gurevich EV Gurevich VV. Receptor-Arrestin Interactions: The Gpcr Perspective. Biomolecules (2021) 11:1–25. 10.3390/biom11020218 PMC7913897 33557162

[52] Ibsen MS Connor M Glass M. Cannabinoid CB1 and CB2 Receptor Signaling and Bias. Cannabis Cannabinoid Res (2017) 2:48–60. 10.1089/can.2016.0037 28861504 PMC5436336

[53] Morales P Goya P Jagerovic N. Emerging Strategies Targeting CB2 Cannabinoid Receptor: Biased Agonism and Allosterism. Biochem Pharmacol (2018) 157:8–17. 10.1016/j.bcp.2018.07.031 30055149

